# Oral Toxicokinetics, Tissue Distribution, and 28-Day Oral Toxicity of Two Differently Manufactured Food Additive Silicon Dioxides

**DOI:** 10.3390/ijms23074023

**Published:** 2022-04-05

**Authors:** Na-Kyung Yoo, Su-Min Youn, Soo-Jin Choi

**Affiliations:** Division of Applied Food System, Major of Food Science & Technology, Seoul Women’s University, Seoul 01797, Korea; iko0105@swu.ac.kr (N.-K.Y.); smyoun@swu.ac.kr (S.-M.Y.)

**Keywords:** synthetic amorphous silica, food additives, toxicokinetics, tissue distribution, 28-d oral toxicity, fate

## Abstract

(1) Background: Synthetic amorphous silica (SAS) is widely used as a food additive and contains nano-sized particles. SAS can be produced by fumed and precipitated methods, which may possess different physiochemical properties, toxicokinetics, and oral toxicity. (2) Methods: The toxicokinetics of fumed SAS and precipitated SAS were evaluated following a single-dose oral administration in rats. The tissue distribution and fate of both SAS particles were assessed after repeated oral administration in rats for 28 d, followed by recovery period for 90 d. Their 28-d repeated oral toxicity was also evaluated. (3) Results: Precipitated SAS showed higher oral absorption than fumed SAS, but the oral absorption of both SAS particles was low (<4%), even at 2000 mg/kg. Our tissue-distribution study revealed that both SAS particles, at a high dose (2000 mg/kg), were accumulated in the liver after repeated administration for 28 d, but the increased concentrations returned to normal levels at 29 d, the first day of the recovery period. A higher distribution level of precipitated SAS than fumed SAS and decomposed particle fates of both SAS particles were found in the liver at 28 d. No significant toxicological findings were observed after 28-d oral administration, suggesting their low oral toxicity. (4) Conclusions: Different manufacturing methods of SAS can, therefore, affect its oral toxicokinetics and tissue distribution, but not oral toxicity.

## 1. Introduction

Food-additive silicon dioxide (SiO_2_), also known as synthetic amorphous silica (SAS), is used as anti-caking, thickening, and encapsulation agents in the food industry [1,2]. It is a natural compound found in plant-based foods, such as oats, barley, and rice. SAS, including fumed silica and hydrated silica (precipitated silica, silica gel, and hydrous silica), is used as E 551 in the European Union [3]. Among various additive forms of SAS, fumed silica and precipitated silica are the ones that are most widely applied to processed foods as directive additives. Fumed SAS, namely pyrogenic SAS, is produced in a flame at 3000 °C from pyrolysis of silicon tetrachloride or from quartz sand [4,5]. Precipitated SAS is manufactured by precipitation from a solution containing silicate salts under acidic conditions [6,7].

The Scientific Committee on Food established an acceptable daily intake (ADI) of silicon dioxide as ‘ADI not specified’, meaning that the total daily intake of silicon dioxide does not represent a hazard to human health based on toxicological and clinical data at its usage levels [8]. Many studies have demonstrated that food-additive SAS can be used without concerns about its potential toxicity [9,10,11]. However, nanoparticles (NPs) ranged from 1 to 100 nm were reported to be present in food-additive SAS [12,13,14]. Recently, the European Food Safety Authority (EFSA) considered E 551 as intentionally produced nano-sized aggregated particles [3]. In this regard, the EFSA suggests that the toxicity of E 551 is required to be re-evaluated. Recent some research showed the potential toxicity of SAS in terms of inflammation of the intestinal wall and neurotoxicity [15,16,17,18]. Contradictory in vivo toxicity results were also reported, showing the low oral toxicity of SAS [18,19,20,21].

Our previous report demonstrated that the solubility and biological fate of food-additive SAS were dependent on manufacturing methods and biological environments, which were highly affected by aggregate formation under biological conditions [22]. Precipitated SAS was less aggregated than fumed SAS in cell culture medium and the gastrointestinal (GI) fluid, resulting in high in vitro cellular uptake, intestinal transport using in vitro epithelial barrier models, and tissue distribution level after a single-dose oral administration in rats compared with fumed SAS [22]; the major fate of SAS was determined to be particle form in human intestinal cells and slowly decomposed into ions during intestinal transport. After a single-dose oral administration in rats, SAS was primarily present as particles in the gastric fluid, but it was mostly in ionic form in the liver, and then only a decomposed ionic form was detected in the kidney.

In this study, oral toxicokinetics of two differently manufactured SAS particles, fumed SAS and precipitated SAS, were evaluated after a single-dose administration in rats. The tissue distribution and oral toxicity of both SAS particles were assessed following 28-d repeated administration in rats to investigate the relation between the toxicokinetics and oral toxicity of SAS particles, depending on manufacturing methods. The fates of SAS in the tissues were also determined by transmission electron microscopy (TEM)–energy dispersive X-ray spectroscopy (EDS) analysis.

## 2. Results and Discussion

### 2.1. Physicochemical Properties

The constituent particle sizes and shapes of fumed SAS and precipitated SAS were analyzed by scanning electron microscopy (SEM) and TEM. The results show that the constituent particles sizes of the former and the latter were 14 ± 4 nm and 16 ± 4 nm, respectively, without significant difference (*p* > 0.05), but high aggregate formation was observed in both cases (Supplementary Figure S1). Thus, both SAS particles can be classified as NPs in terms of constituent particle size. Irregular round shapes were observed for both particles, but more smooth edges were found for fumed SAS. X-ray diffraction (XRD) patterns of both particles showed broad diffraction peaks between 15 and 32 degrees, centered at 2-theta = around 22 degrees, which are typical for amorphous silica (Supplementary Figure S2A) [23]. The hydrodynamic diameters (375 ± 1 nm) of precipitated SAS in distilled water (DW) were significantly larger than those (156 ± 1 nm) of fumed SAS (Supplementary Figure S2B), suggesting high aggregate formation of the former in DW. It is worth noting that precipitated SAS was determined to be less aggregated than fumed SAS in cell culture minimum essential medium and three consecutive steps of the GI fluid (Figure S2B) [22], indicating different aggregate states of SAS depending on the biological environment. 

The Zeta potentials of fumed SAS and precipitated SAS were −24 ± 1 mV and −22 ± 1 mV—without significant difference (*p* > 0.05) (Supplementary Figure S2C). The specific surface areas, measured by using the Brunauer–Emmett–Teller (BET) method, were 177 ± 1 m^2^/g and 167 ± 1 m^2^/g for fumed SAS and precipitated SAS, respectively, demonstrating a significantly high BET value for the former (Supplementary Figure S2D). The high BET value of fumed SAS was surely associated with its small hydrodynamic diameters in DW (Supplementary Figure S2B). These results suggest that the hydrodynamic diameters and specific surface areas of SAS could be affected by the manufacturing methods.

### 2.2. Plasma Concentration–Time Profiles

**Figure 1:**
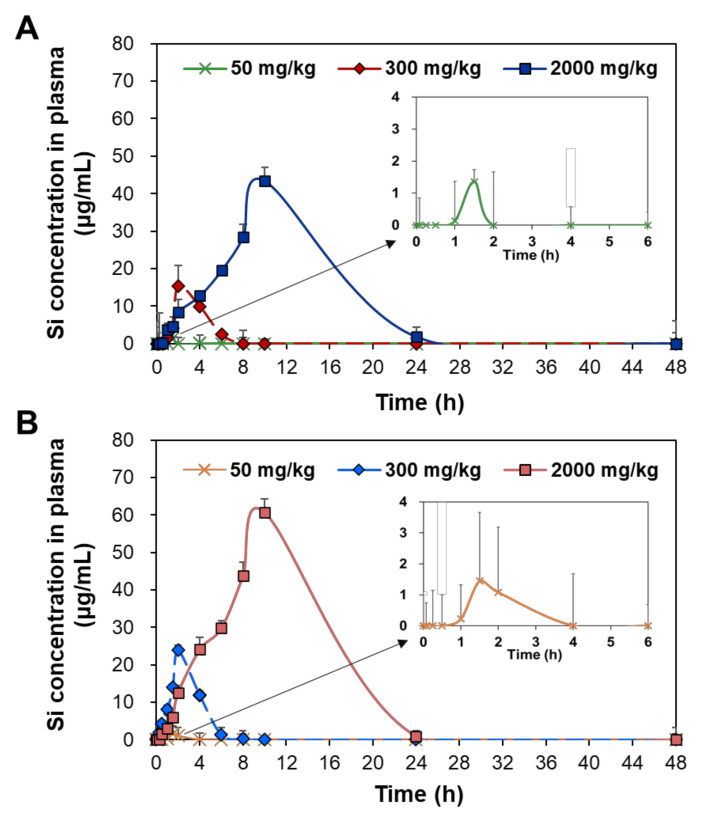
Plasma concentration–time curves of (**A**) fumed SAS and (**B**) precipitated SAS after a single-dose oral administration of three different doses (50, 300, and 2000 mg/kg) in rats.

Toxicokinetics reflects how the body treats toxic materials in terms of absorption, distribution, metabolism, and excretion and can be predicted by the plasma concentration–time curves after administration. The plasma concentration–time profiles of two differently manufactured SAS particles were evaluated after a single-dose oral administration in rats. Three different doses (50, 300, and 2000 mg/kg) were administered based on its no-observed-adverse-effect level (NOAEL) value of 2000 mg/kg and low oral absorption [24,25]. Figure 1 demonstrates that the plasma concentration–time profiles increased as administered doses increased. The plasma curves rapidly deceased within 4 and 8 h when the low dose (50 mg/kg) and medium dose (300 mg/kg) were administered, respectively, but a high peak concentration and slow decrease in the curves were found at high dose (2000 mg/kg). In all cases, precipitated SAS showed higher plasma concentration–time curves and peak concentrations than fumed SAS. 

The toxicokinetic parameters were calculated based on the curves (Figure 1) and are presented in Table 1. At the low dose (50 mg/kg), no significant differences in maximum concentration (C_max_), time to maximum concentration (T_max_), half-life (T_1/2_), and mean residence time (MRT) were found between fumed SAS and precipitated SAS (*p* > 0.05). However, the AUC values and oral absorption (%) were significantly higher for precipitated SAS than for fumed SAS. The apparent clearance (Cl/F) values of both SAS particles were not obtained, probably because of their low oral absorption levels. At medium and high doses (300 and 2000 mg/kg), significantly high C_max_, T_1/2_, MRT, AUC, and oral absorption values for the precipitated SAS were found compared with those of the fumed SAS (*p* < 0.05). No statistical differences in T_max_ values were found between the two materials (*p* > 0.05). The Cl/F values were higher for the fumed SAS than for the precipitated SAS. The oral absorptions of the fumed SAS and precipitated SAS at the high dose (2000 mg/kg) were 3.1% and 3.9%, respectively.

All toxicokinetic results suggest that the oral absorption of the precipitated SAS was significantly higher than it was for the fumed SAS, thereby meaning that the former remained longer in the body. In other words, fumed SAS can be more rapidly eliminated from the body than precipitated SAS, as demonstrated by lower T_1/2_ and MRT values and higher Cl/F values. In all cases, both SASs could be cleared from the bloodstream within 21 h, as indicated by the MRT values. Hence, the oral toxicokinetics of SAS can differ from manufacturing methods. The high oral absorption of the precipitated SAS can be explained by its small hydrodynamic diameters in the GI fluids (Supplementary Figure S2B). NPs were reported to be more massively absorbed into the bloodstream than bulk-sized particles [26,27]. Our previous study also showed a higher intestinal transport amount of the precipitated SAS than the fumed SAS, using in vitro intestinal barrier models [22]. It should be noted that the oral absorption of SAS was less than 4%, even at 2000 mg/kg, and this finding is also in good agreement with the previous report [28]. The low oral absorption and rapid clearance of SAS also suggest its low potential for tissue accumulation and organ burden. On the other hand, SAS particles seem to be absorbed in both particle and ionic forms regardless of manufacturing methods, considering their maximum oral absorption up to 3.9% (Table 1) and in vitro and ex vivo solubilities of 1.8–2.8% in the GI fluids [22].

### 2.3. Tissue Distribution

The tissue distribution and possible organ burden were assessed after repeated oral administration in rats for a consecutive 28 d, since the oral absorption of SAS is low after a single-dose oral administration (Figure 1 and Table 1). The tissue-distribution study was further carried out during the recovery period for 90 d, following the 28-d repeated oral administration; thus, organ burden was investigated for total 118 d. This experimental design was aimed to answer the question as to whether SAS with constituent particle sizes of less than 100 nm (Supplementary Figure S1) can be accumulated in the specific organ after repeated exposure despite its low oral bioavailability. Recovery period was set to assess possible delayed accumulation and elimination from the body. The liver, kidney, spleen, lymph node, stomach, and small intestine were selected for the main tissue distribution study, because NPs tend to be accumulated in these organs after oral exposure [29,30,31]. The total Si levels in the tissues were determined by using inductively coupled plasma–atomic emission spectroscopy (ICP–AES).

**Figure 2:**
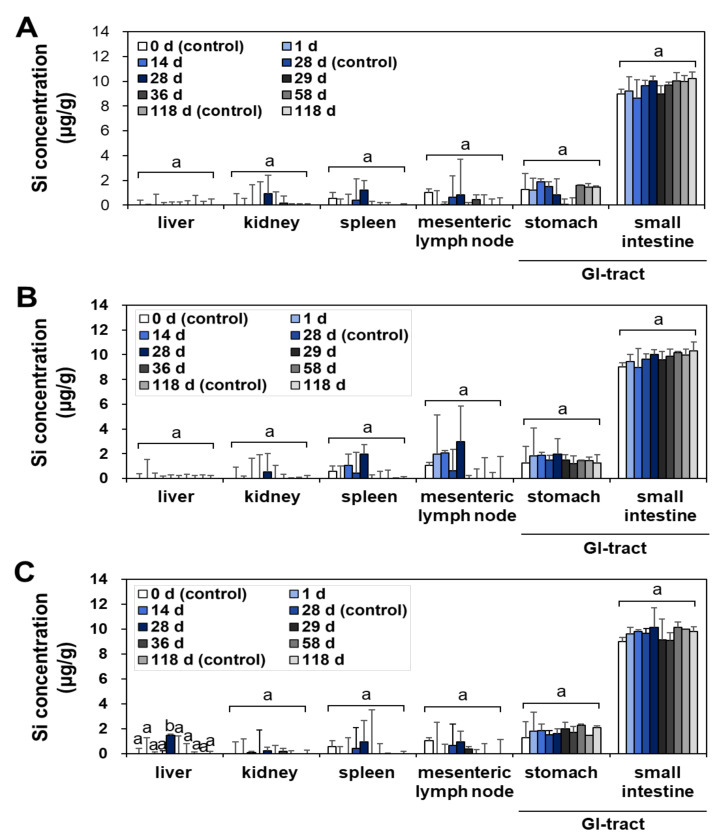
Tissue distribution of fumed SAS at (**A**) 50 mg/kg, (**B**) 300 mg/kg, and (**C**) 2000 mg/kg after 28-d repeated oral administration in rats, followed by 90-d recovery period. Different lower-case letters (a,b) indicate significant differences among different time points of the same tissue (*p* < 0.05).

**Figure 3:**
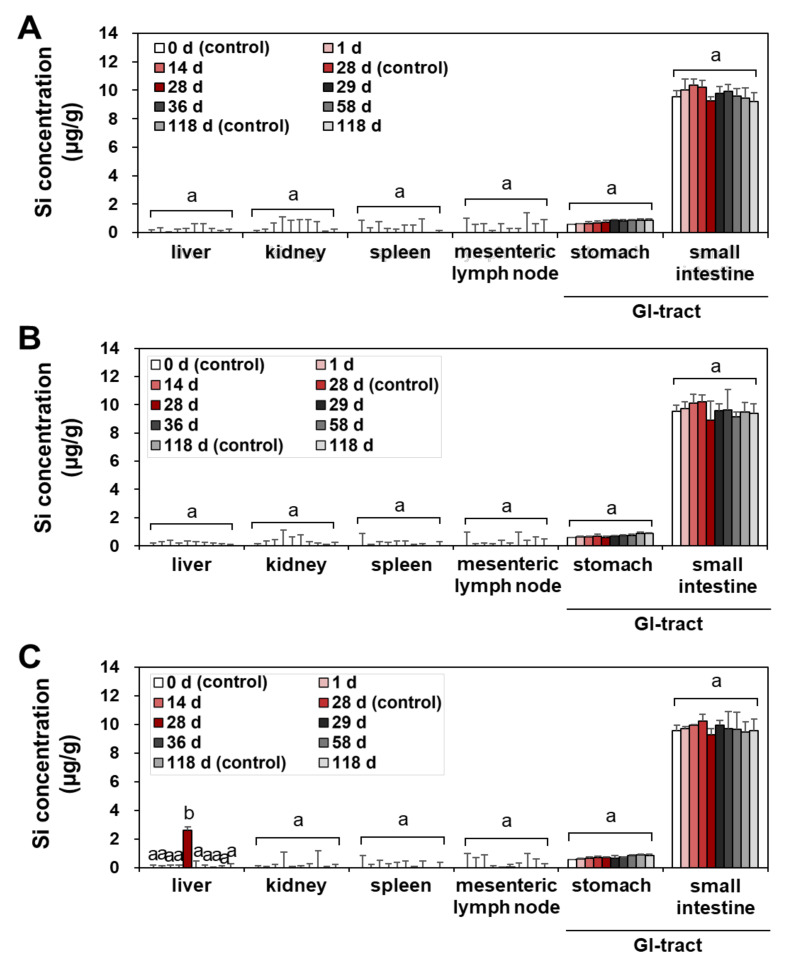
Tissue distribution of precipitated SAS at (**A**) 50 mg/kg, (**B**) 300 mg/kg, and (**C**) 2000 mg/kg after 28-d repeated oral administration in rats, followed by 90-d recovery period. Different lower-case letters (a,b) indicate significant differences among different time points of the same tissue (*p* < 0.05).

No significant accumulation was found in all organs at the low (50 mg/kg) (Figure 2A and Figure 3A) and medium (300 mg/kg) (Figure 2B and Figure 3B) doses of fumed SAS and precipitated SAS, compared with the non-treated control group during repeated administration for 28 d, followed by a recovery period for 90 d (Figure 2 and Figure 3). However, significantly increased Si levels were detected in the liver at 28 d after oral administration of both fumed SAS and precipitated SAS at the high dose (2000 mg/kg), and then the increased Si concentrations returned to normal levels at 29 d, the first day of the recovery period (Figure 2C and Figure 3C). No significant accumulation was found in the other organs.

**Figure 4:**
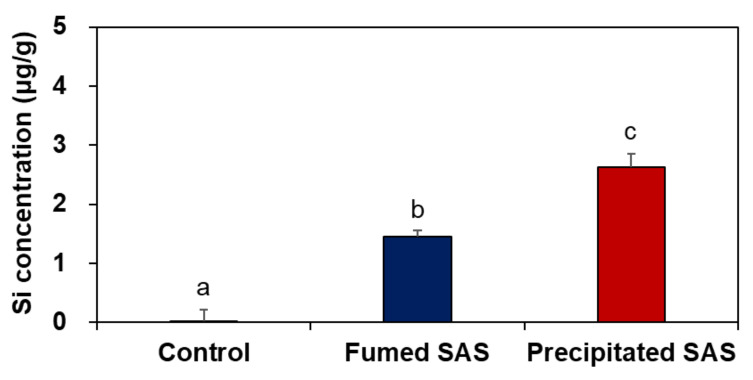
Tissue accumulation of SAS particles in the liver at 28 d after 28-d repeated oral administration at 2000 mg/kg in rats. Different lower-case letters (a–c) indicate significant differences among non-treated control, fumed SAS-treated, and precipitated SAS-treated groups (*p* < 0.05).

When the tissue accumulation in the liver at 28 d for the high dose (2000 mg/kg) was compared, a significantly higher Si level was found for the precipitated SAS than for the fumed SAS (Figure 4) (*p* < 0.05), although increased Si levels were marginal (1.5–2.6 μg/g). On the other hand, no increased accumulation was found in the brain and lung by two differently manufactured SAS particles (Supplementary Figure S3). The results clearly show that the liver is a main target organ for tissue accumulation of SAS, regardless of manufacturing methods. However, precipitated SAS could more massively distribute to the liver than fumed SAS, and this is surely related to smaller hydrodynamic diameters of the former than the latter in the GI fluids (Supplementary Figure S2B). Moreover, the high oral absorption of the precipitated SAS compared with the fumed SAS (Figure 2 and Table 1) also supports this result. It was reported that small-sized particles can be easily taken up by cells and more massively absorbed into the body [32,33]. It is worth noting that increased Si concentrations in the liver by a high dose of both SAS particles were low (1.5–2.6 μg/g) and returned to normal basal levels at 29 d, just after stopping administration, thus suggesting relatively easy elimination of the absorbed SAS particles from the body. These results are consistent with the report showing low oral absorption and low potential for organ burden of food-additive silicon dioxide [3]. Further study is required to investigate excretion pathways, kinetics, and particle/ionic fate of SAS after oral intake.

### 2.4. Tissue Distribution Fates

**Figure 5:**
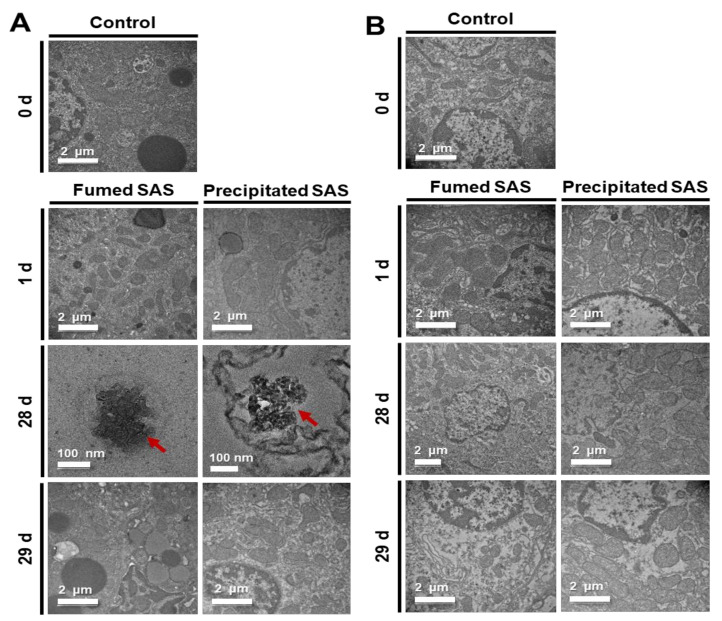
Transmission electron microscopy (TEM) images of the (**A**) livers and (**B**) kidneys after 28-d repeated oral administration of fumed SAS and precipitated SAS at 2000 mg/kg in rats, followed by recovery period. Arrows in red color indicate SAS particles found in the livers.

The fates of fumed SAS and precipitated SAS in the tissues were determined by TEM analysis at 1, 28, and 29 d after repeated oral administration of high dose (2000 mg/kg), followed by recovery period. The liver was selected to determine their tissue fates based on the tissue distribution results (Figure 2, Figure 3 and Figure 4). The kidney was also included to examine possible excretion of absorbed SAS by this organ. Figure 5 shows that decomposed particle forms were detected in the liver at 28 d, but the particles were not observed in the liver at 29 d, the first day of the recovery period. Almost decomposed particles were observed in the liver treated with fumed SAS, whereas relatively intact particle forms were found by precipitated SAS. No particles were detected in the kidney. Taken together, absorbed SAS can distribute in particle forms and be slightly decomposed in the liver during metabolic process, and then probably be eliminated from the kidney in ionic forms. The manufacturing methods of SAS can also affect its fate in the tissue, related to the extent of oral absorption (Figure 1 and Table 1). It seems that more time is required to decompose precipitated SAS with high oral absorption. This result is in good agreement with the tissue fates of colloidal silicon dioxide NPs, demonstrating their particle fate only in the liver [34].

**Figure 6:**
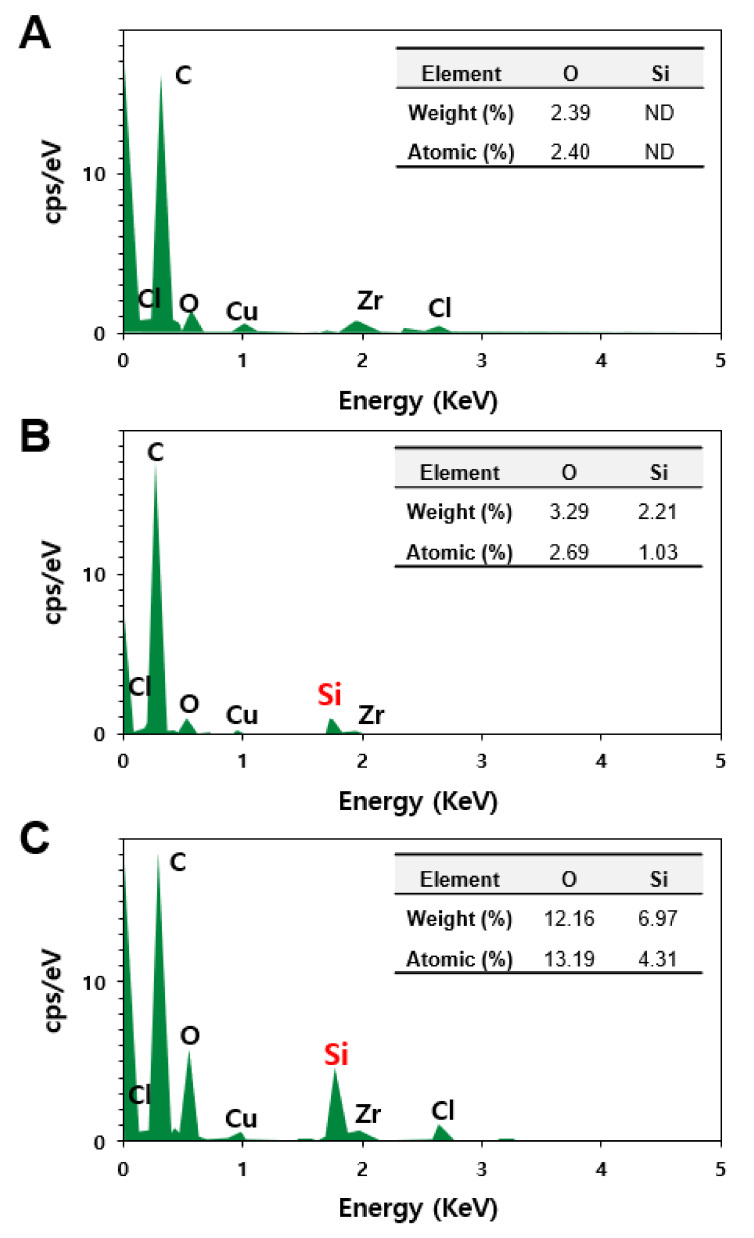
Energy dispersive X-ray spectroscopy (EDS) analysis of particle forms observed in the rat livers at 28 d in non-treated control (**A**) and after 28-d repeated oral administration of (**B**) fumed SAS and (**C**) precipitated SAS at 2000 mg/kg. ND, not detectable.

An energy dispersive X-ray spectroscopy (EDS) analysis was further carried out for the liver, where decomposed particles were observed. EDS is an analytical method used in conjunction with electron microscopy for elemental analysis or chemical characterization. Figure 6 shows that no Si was detected in the non-treated control rat liver (Figure 6A), whereas significantly elevated Si levels were found in the livers treated with fumed SAS (Figure 6B) and precipitated SAS (Figure 6C). When atomic levels (%) of O and Si, two elemental components of SAS (shown in the inserted tables in Figure 6) were compared, we observed that the O levels were higher in the livers treated with both SASs than that in the control, and a remarkably increased Si level was detected in precipitated SAS-treated liver compared to fumed-SAS-treated liver. Hence, the presence of Si in the rat livers administered fumed SAS and precipitated SAS were confirmed, showing a high Si level in the liver treated with the precipitated SAS compared to the fumed SAS. This result also supports the tissue distribution (Figure 4) and tissue fate (Figure 5) of both SAS particles (Figure 6).

### 2.5. Twenty-Eight-Day Repeated Oral Toxicity

**Figure 7:**
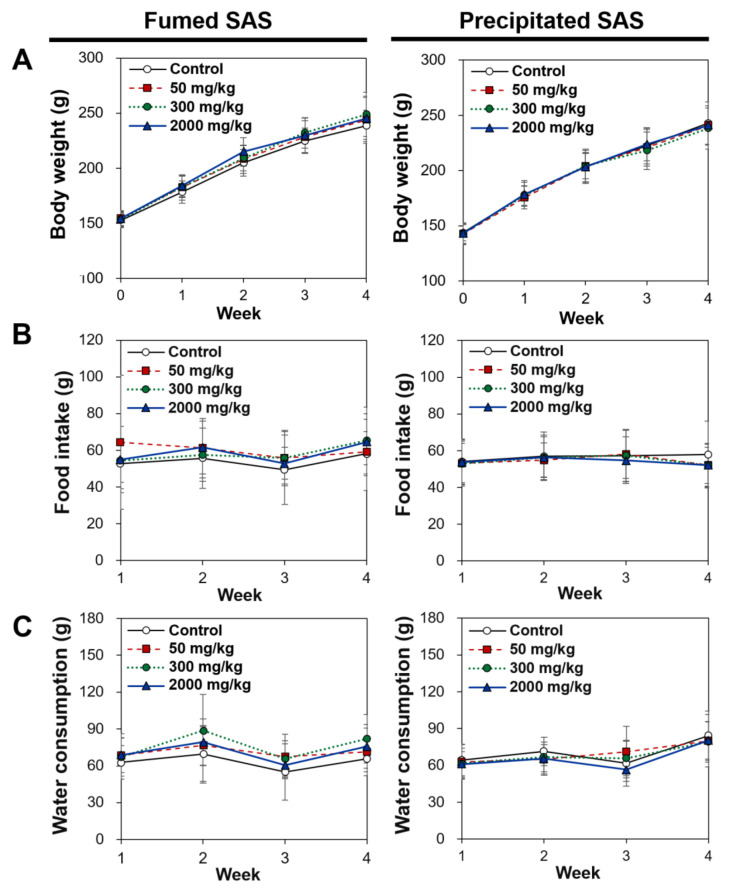
Changes in (**A**) body weight, (**B**) food intake, and (**C**) water consumption after 28-d repeated oral administration of fumed SAS and precipitated SAS in rats. No significant differences among control, fumed SAS-treated, and precipitated SAS-treated groups were found (*p* > 0.05).

The general toxicological findings were observed during repeated oral administration of three different doses for 28 d. As shown in Figure 7, no significant differences in food intake, water consumption, and body-weight gains were found in the rats administered both SAS particles compared with the non-treated control group. No abnormal behaviors were observed during the administration period. Necropsy findings also suggest no significant changes in rats administered both particles, suggesting the low oral toxicity potential of SAS.

A further analysis of the absolute and relative organ weights, as well as hematological and serum biochemical parameters, was carried out in rats administered the high dose (2000 mg/kg) for 28 d, because the NOAEL value for silicon dioxide was reported to be more than 2000 mg/kg in the previous studies [24,25,35] and the oral absorption of both particles was low (less than 4%) in the present study (Figure 1 and Table 1). Table 2 and Table 3 demonstrate that no significant differences in all absolute and relative organ weights of the rats were found among the control, fumed SAS-treated, and precipitated SAS-treated groups (*p* > 0.05).

The hematological and coagulation time values of rats arere presented in Table 4, showing significant differences in the white blood cell (WBC), lymphocyte (LY), monocyte (MO), and eosinophil (EO) values of the rats administered precipitated SAS, compared with those of the non-treated control, based on differential leucocyte counts as 10^3^ cells/µL (*p* < 0.05). However, these values were not significantly different when differential leucocyte counts were calculated as % (*p* > 0.05). No other values of fumed SAS and precipitated SAS were statistically different from those of the non-treated control group (*p* < 0.05).

All serum biochemical values of rats administered fumed SAS and precipitated SAS were statistically the same as the values of the non-treated control (Table 5). It is likely that both SAS particles did not exhibit oral toxicity even after repeated administration of 2000 mg/kg for 28 d.

**Figure 8:**
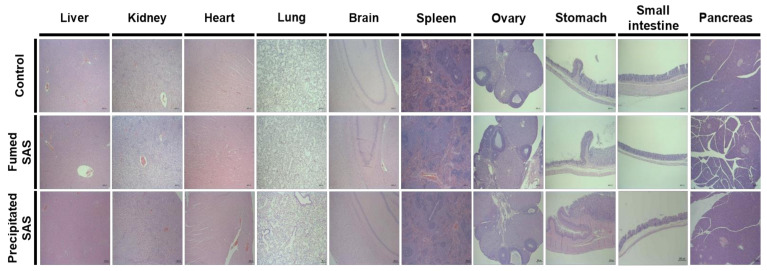
Representative histopathological images of organs (liver, kidney, heart, lung, brain, spleen, ovary, stomach, small intestine, and pancreas) after 28-d repeated oral administration of fumed SAS and precipitated SAS in rats. Images were magnified at 50×. No significant abnormal lesions were observed in non-treated control, fumed SAS-treated, and precipitated SAS-treated groups.

The histopathological examination revealed that periportal vacuolation in the liver and minimal tubular basophilia in the kidney were found in one rat in the non-treated control group after 28-d repeated oral administration of 2000 mg/kg in rats (Table 6). Minimal inflammatory cell foci in the liver, slight tubular basophilia, medullary cyst, cortical scar in the kidney, and squamous cyst in the stomach were observed in the rats administered the SAS particles (Table 6). Figure 8 demonstrates representative images of all organs of non-treated control rats and rats administered fumed SAS or precipitated SAS. It is worth noting that the abnormal histopathological findings observed for SAS particles were minimal and slight, and they are often found in background lesions [36,37]. Some minimal lesions were also observed in the control group. Moreover, no significant toxicological findings were found in terms of organ weight, hematological, coagulation time, and serum biochemical values (Table 2, Table 3, Table 4 and Table 5). It is strongly likely that these histopathological findings were not associated with both of the SAS particles administered. It can be, therefore, concluded that fumed SAS and precipitated SAS did not cause oral toxicity after repeated administration for 28 d, and the NOAEL values of both particles were more than 2000 mg/kg. 

## 3. Materials and Methods

### 3.1. Materials

Food-grade fumed SAS (AEROSIL^®^ 200F) and precipitated SAS (SIPERNAT^®^ 22S) were obtained from Evonik Industries AG (Essen, NRW, Germany). SAS particles were suspended in DW to designed concentrations (5, 30, and 200 mg/mL) and stirred for 30 min, followed by sonication for 15 min (bath sonicator, 160 Watts, Bransonic 5800, Branson Ultrasonics, Danbury, CT, USA) on the day of experiments.

Nitric acid (HNO_3_) was provided from Junsei Chemical Co., Ltd. (Tokyo, Japan). Hydrogen fluoride (HF), sodium hydroxide (NaOH), and hydrogen peroxide (H_2_O_2_) were purchased from Samchun Pure Chemical Co., Ltd. (Pyeongtaek, Gyeonggi-do, Korea). Si standard solution was obtained from Sigma-Aldrich (St. Louis, MO, USA). Isoflurane was acquired from Hana Pharm Co., Ltd. (Seoul, Korea).

### 3.2. Animals

Five-week-old female Sprague-Dawley (SD) rats were supplied from Koatech Co. (Pyeongtaek, Gyeonggi-do, Korea) for toxicokinetic study that was performed at Seoul Women’s University. Five-week-old female SD rats were provided from G-Bio (Gwangju, Korea) for tissue distribution and oral toxicity following 28-d repeated administration performed at the Korea Testing & Research Institute (KTR, Jeollanam-do, Korea), a good laboratory practice institution. Experimental animals were maintained in a clean animal rack controlled at 22 ± 3 °C with relative humidity of 50 ± 10% and a light/dark cycle of 12 h. Water and commercial standard feed were given ad libitum. All animal experiments were performed with approval in accordance with the guidelines for the Institutional Animal Care and Use Committee (IACUC) of the Seoul Women’s University (SWU IACUC-2020A-2) and the KTR IACUC (IAC2020-2120; IAC2021-0417).

### 3.3. Microwave Digestion and ICP–AES Analysis

The levels of SAS in biological samples were quantified by measuring total Si concentrations, using ICP–AES (JY2000 Ultrace, HORIBA Jobin Yvon, Longjumeau, France) after microwave digestion, as described in our previous study [22,38]. Briefly, 0.2 g of the sample was transferred to a perfluoroalkoxy alkane vessel, and 6 mL of 70% HNO_3_ and 1 mL of 40% HF were added. The samples were digested at 1600 W for a total of 56 min, with the following temperature program: from 25 to 120 °C in 15 min, from 120 to 160 °C in 10 min, and from 160 to 210 °C in 30 min, and then hold for 1 min, using a microwave digestion system (ETHOS EASY, Milestone SrL, Sorisole, Italy). After digestion, the samples were cooled to room temperature, diluted with distilled deionized water, and gently shaken. The prepared samples were analyzed by using ICP–AES (JY2000 Ultrace, HORIBA Jobin Yvon) at 1000 W (radiofrequency power) with a plasma gas flow of 12 L/min.

### 3.4. Plasma Concentration–Time Profiles

Six female SD rats per group were orally administered a single dose of 50, 300, or 2000 mg/kg of fumed SAS and precipitated SAS by gavage, respectively. Only females, as they are generally considered to be more sensitive to toxic materials, were selected for toxicokinetic study, because no evidence on significant sex-related differences in toxicity and toxicokinetics of silicon dioxide was reported [34]. Blood samples were collected from the tail vein of rats at 0, 0.08, 0.25, 0.5, 1, 1.5, 2, 4, 6, 8, 10, 24 and 48 h post-administration. The plasma samples were obtained after centrifugation (3000× *g*, 1 min, 4 °C) of the blood samples. The total Si concentrations in the plasma were determined after digestion, as described in Section 3.3. The following toxicokinetic parameters were calculated by using a pharmacokinetic modeling program (version 1.03.35, APL, Eden Prairie, MN, USA): C_max_, T_max_, T_1/2_, MRT, Cl/F, and AUC. Oral absorption (%) was calculated based on AUC values and administered doses. 

### 3.5. Tissue Distribution after 28-d Repeated Oral Administration

Five female rats per group were administered three different doses (50, 300, or 2000 mg/kg) of fumed SAS and precipitated SAS via oral gavage for 28 d, followed by a recovery period for 90 d. An equivalent volume of DW was also administered in rats as a control group. To determine tissue distribution and accumulation of each particle, time points were designed as follows: (1) the control group at 0, 28, and 118 d; (2) SAS-administered groups at 1, 14, 28, 29, 36, 58, and 118 d. At time points designated, the whole blood and tissues (liver, kidney, spleen, mesenteric lymph node, stomach, small intestine, brain, and lung) were collected after anesthetizing and euthanizing the rats with isoflurane solution. All samples were stored at −80 °C before analysis. The tissues were rinsed with saline and homogenized by chopping with stainless steel scissors, and then, total Si levels in the tissues were quantified as described in Section 3.3.

### 3.6. Twenty-Eight-Day Repeated Oral Toxicity

Five female SD rats per group were administered a single dose (2000 mg/kg) of fumed SAS or precipitated SAS via oral gavage for 28 d. A control group received an equivalent volume of DW. During the experimental period, changes in body weight, food intake, and water consumption were recorded, and abnormal symptoms and behaviors were observed daily before and after administration. After repeated administration for 28 d, the rats were fasted for more than 18 h and anesthetized with isoflurane solution. The whole blood was then collected from the abdominal aorta. Hematological parameters and blood coagulation time values were analyzed by using a hematology analyzer (ADVIA 2120i, Siemens, Germany) and coagulometer (ACL Elite Pro, Instrumentation Laboratory, Bedford, MA, USA), respectively. Serum biochemical parameters were evaluated by using biochemistry analyzer (TBA-120FR, Toshiba, Tokyo, Japan).

After euthanasia, the external surface, all orifices, cranial cavity, thoracic and abdominal cavities, and organs of the rats were visually inspected. Absolute and relative (organ weight to body weight ratios, %) organ weights were assessed. For histopathological examination, the tissues were fixed in 10% neutral buffered formalin, followed by dehydration, paraffin penetration, embedding, and sectioning. Then the tissue sections were stained with hematoxylin and eosin for histopathological examination.

### 3.7. TEM–EDS Analysis

To confirm the presence of SAS particles in the rat liver and kidney, the tissue samples were fixed in 2% glutaraldehyde and 2% paraformaldehyde in phosphate buffer (pH 7.4) for 1 h at 4 °C. The samples were post-fixed in osmium tetroxide for 40 min at 4 °C and dehydrated in a graded series of ethanol. Then the samples were treated with a graded propylene oxide series, embedded into Epon 812 resin, and sectioned in 80 nm by using ultra-microtome. Finally, the tissue sections were placed on copper grid and stained with uranyl acetate and lead citrate. The images and chemical compositions were obtained at an accelerating voltage of 200 kV, using TEM (JEM-2100F, JOEL, Tokyo, Japan) equipped with EDS, at the Korea Basic Science Institute (KBSI, Chuncheon, Gangwon-do, Korea).

### 3.8. Statistical Analysis

Data were presented as means ± standard deviations. Statistical significance was determined by using the SPSS (version 19.0, SPSS Inc., Chicago, IL, USA) or SAS (version 9.4, SAS Institute Inc., Cary, NC, USA). A one-way ANOVA test was performed for data on oral toxicokinetics, body weight, feed intake, and water consumption. A *t*-test was performed for organ weights and hematological and serum biochemical values. Statistical significance was accepted for *p*-values of <0.05.

## 4. Conclusions

The physicochemical properties, oral toxicokinetics, tissue distribution, and oral toxicity of two differently manufactured food-additive SAS particles, fumed SAS and precipitated SAS, were evaluated. The precipitated SAS had higher oral absorption than the fumed SAS after a single-dose administration in rats, probably because of smaller hydrodynamic diameters of the former than the latter in the GI fluids. Both SAS particles were determined to be accumulated only in the rat liver at 28 d, following repeated oral administration for 28 d, but the organ accumulation returned to normal basal level at 29 d, the first day of the recovery period. Precipitated SAS was highly accumulated in the liver compared with fumed SAS, attributed to the higher oral absorption of the former than the latter. Both SAS particles were found in decomposed particle forms in the liver, but not in the kidney, suggesting that they could be taken up by the tissue in particle forms, slowly decomposed in the liver during metabolic process, and then probably eliminated from the kidney in ionic forms. The repeated oral toxicity study for 28 d revealed no significant toxicological findings caused by both SAS particles. Hence, the NOAEL values of SAS particles were more than 2000 mg/kg, suggesting their low oral toxicity. It can be, therefore, concluded that different manufacturing methods of SAS can affect oral toxicokinetics and tissue distribution, but not oral toxicity. An investigation on excretion pathways, kinetics, fates, and chronic oral toxicity of SAS is required to be performed. 

## Figures and Tables

**Table 1:** Toxicokinetic parameters and oral absorption (%) after a single-dose oral administration of three different doses (50, 300, and 2000 mg/kg) of fumed SAS and precipitated SAS in rats.

Toxicokinetic Parameters	Fumed SAS			Precipitated SAS		
	50 mg/kg	300 mg/kg	2000 mg/kg	50 mg/kg	300 mg/kg	2000 mg/kg
C_max_ (μg/mL)	1.4 ± 0.4 ^A,a^	15.3 ± 5.5 ^A,b^	43.4 ± 3.6 ^A,c^	1.5 ± 2.2 ^A,a^	23.9 ± 1.2 ^B,b^	60.7 ± 3.7 ^B,c^
T_max_ (h)	1.5 ± 0.0 ^A,a^	2.0 ± 0.0 ^A,b^	10.0 ± 0.0 ^A,c^	1.5 ± 0.0 ^A,a^	2.0 ± 0.0 ^A,b^	10.0 ± 0.0 ^A,c^
T_1/2_ (h)	1.7 ± 0.2 ^A,a^	3.3 ± 0.2 ^A,b^	16.6 ± 0.2 ^A,c^	1.8 ± 0.1 ^A,a^	3.7 ± 0.1 ^B,b^	18.2 ± 0.5 ^B,c^
MRT (h)	1.8 ± 0.2 ^A,a^	3.8 ± 0.2 ^A,b^	18.6 ± 0.3 ^A,c^	2.0 ± 0.2 ^A,a^	4.2 ± 0.2 ^B,b^	20.7 ± 1.2 ^B,c^
Cl/F (mL/h)	ND	544.1 ± 9.5 ^A,a^	331.9 ± 11.1 ^A,b^	ND	352.2 ± 17.9 ^B,a^	20.7 ± 1.2 ^B,c^
AUC (h × μg/mL)	0.7 ± 0.1 ^A,a^	50.3 ± 8.8 ^A,b^	577.4 ± 55.1 ^A,c^	1.6 ± 0.1 ^B,a^	85.3 ± 4.5 ^B,b^	727.6 ± 19.2 ^B,c^
Absorption (%)	0.2 ± 0.0 ^A,a^	1.8 ± 0.3 ^A,b^	3.1 ± 0.3 ^A,c^	0.4 ± 0.0 ^B,a^	2.9 ± 0.2 ^B,b^	3.9 ± 0.1 ^B,c^

Different upper-case letters (^A,B^) indicate significant differences between fumed SAS and precipitated SAS (*p* < 0.05). Different lower-case letters (^a–c^) indicate significant differences among three different doses (50, 300, and 2000 mg/kg) of the same SAS particles (*p* < 0.05). C_max_, maximum concentration; T_max_, time to maximum concentration; T_1/2_, half-life; MRT, mean residence time; Cl/F, apparent clearance; AUC, area under the plasma concentration–time curve; ND, not determined.

**Table 2:** Absolute organ weights of rats after 28-d repeated oral administration of fumed SAS and precipitated SAS.

Group	Liver (g)	Kidney (g)	Spleen (g)	Heart (g)	Brain (g)	Lung (g)	Thymus (g)	Submandibular Lymph Node (g)	Mesenteric Lymph Node (g)	Ovary (g)
Control	7.2 ±0.3	2.3 ±0.2	0.5 ±0.1	1.0 ±0.1	2.1 ±0.1	1.3 ±0.4	0.7 ±0.1	0.9 ±0.4	0.3 ±0.1	0.1 ±0.0
Fumed SAS	7.4 ±0.8	2.4 ±0.5	0.6 ±0.1	1.0 ±0.1	2.1 ±0.1	1.2 ±0.4	0.7 ±0.4	0.6 ±0.2	0.3 ±0.1	0.1 ±0.0
Precipitated SAS	7.3 ±0.3	2.3 ±0.2	0.5 ±0.1	1.1 ±0.1	2.1 ±0.1	1.3 ±0.4	0.7 ±0.1	0.9 ±0.5	0.3 ±0.0	0.1 ±0.1

No significant differences among non-treated control, fumed SAS-treated, and precipitated SAS-treated groups were found (*p* > 0.05). In case of organs with left and right sides, both sides were measured, and the weights were combined.

**Table 3:** Relative organ weights of rats after 28-d repeated oral administration of fumed SAS and precipitated SAS.

Group	Body Weight (g)	Liver (%)	Kidney (%)	Spleen (%)	Heart (%)	Brain (%)	Lung (%)	Thymus (%)	Submandibular Lymph Node (%)	Mesenteric Lymph Node (%)	Ovary (%)
Control	221.7 ±0.1	3.2 ±0.1	1.0 ±0.1	0.2 ±0.0	0.5 ±0.0	0.9 ±0.0	0.6 ±0.2	0.3 ±0.1	0.4 ±0.2	0.1 ±0.0	0.1 ±0.0
Fumed SAS	224.7 ±8.7	3.3 ±0.3	1.1 ±0.2	0.3 ±0.1	0.5 ±0.1	0.9 ±0.0	0.6 ±0.2	0.3 ±0.2	0.3 ±0.1	0.1 ±0.0	0.1 ±0.0
Precipitated SAS	225.5 ±6.0	3.2 ±0.1	1.0 ±0.1	0.2 ±0.0	0.5 ±0.0	0.9 ±0.0	0.6 ±0.2	0.3 ±0.1	0.4 ±0.2	0.1 ±0.1	0.1 ±0.0

No significant differences among non-treated control, fumed SAS-treated, and precipitated SAS-treated groups were found (*p* > 0.05). In case of organs with left and right sides, both sides were measured, and the weights were combined. Relative organ weights were calculated as combined-organ-weight-to-body-weight ratios (%).

**Table 4:** Hematological and coagulation time values of rats after 28-d repeated oral administration of fumed SAS and precipitated SAS.

Group	WBC (10^3^ cells/μL)	Differential Leucocyte Count (10^3^ cells/μL)					Differential Leucocyte Count (%)					RBC (10^6^ cells/µL)	HGB (g/dL)	HCT (%)	MCV (fL)	MCH (pg)	MCHC (g/dL)	Retic (10^9^ cells/L)	Retic (%)	PLT (10^3^ cells/μL)	PT (s)	APTT (s)
		NEs	LYs	MOs	EOs	BAs	NEs	LYs	MOs	EOs	BAs											
**Control**	4.3 ±1.2	0.4 ±0.2	3.7 ±1.1	0.04 ±0.03	0.05 ±0.02	0.0 ±0.0	9.9 ±3.6	87.0 ±3.6	0.9 ±0.3	1.3 ±0.2	0.1 ±0.0	7.3 ±0.3	14.1 ±0.6	42.7 ±2.1	58.3 ±0.6	19.3 ±0.3	33.0 ±0.4	136.3 ±18.2	1.9 ±0.2	844.2 ±509.7	16.1 ±0.6	13.7 ±1.2
**Fumed** **SAS**	4.4 ±1.7	0.7 ±0.5	3.5 ±1.3	0.07 ±0.03	0.08 ±0.04	0.0 ±0.0	16.4 ±5.4	79.6 ±5.7	1.7 ±0.4	1.7 ±0.4	0.1 ±0.0	7.4 ±0.3	14.6 ±0.3	44.0 ±0.9	59.3 ±1.9	19.8 ±0.5	33.3 ±0.3	172.5 ±30.2	2.3 ±0.5	1159.0 ±65.0	16.1 ±0.7	13.4 ±1.7
**Precipitated SAS**	6.6 ±1.4 *	0.7 ±0.7	5.7 ±0.8 *	0.07 ±0.03 *	0.07 ±0.01 *	0.0 ±0.0	9.8 ±7.1	87.8 ±6.1	1.0 ±0.2	1.3 ±0.1	0.1 ±0.0	7.3 ±0.3	14.4 ±0.6	43.4 ±2.1	59.1 ±0.3	19.7 ±0.2	33.2 ±0.7	129.2 ±18.2	1.8 ±0.3	843.8 ±971.8	17.4 ±0.8	14.7 ±1.5

* Indicates significant differences compared with non-treated control group (*p* < 0.05). WBC, white blood cell; NEs, neutrophils; LYs, lymphocytes; MOs, monocytes; EOs, eosinophils; BAs, basophils; RBC, red blood cell; HGB, hemoglobin, HCT, hematocrit; MCV, mean corpuscular volume; MCH, mean corpuscular hemoglobin; MCHC, mean corpuscular hemoglobin concentration; Retic, reticulocyte; PLT, platelet; PT, prothrombin time; APTT, activated partial thromboplastin time.

**Table 5:** Serum biochemical values of rats after 28-d repeated oral administration of fumed SAS and precipitated SAS.

Group	TP (g/dL)	ALB (g/dL)	A/G ratio	T-BIL (mg/dL)	ALP (U/L)	AST (U/L)	ALT (U/L)	CREA (mg/dL)	BUN (mg/dL)	T-CHO (mg/dL)	TG (mg/dL)	GLU (mg/dL)	CA (mg/dL)	IP (mg/dL)	GGT (IU/L)	CK (IU/L)	TBA (µmol/L)	Na^+^ (mmol/L)	K^+^ (mmol/L)	Cl^−^ (mmol/L)	CHE (U/L)
**Control**	5.8 ±0.3	3.8 ±0.2	1.9 ±0.2	0.0 ±0.0	398.0 ±65.7	89.4 ±5.8	29.2 ±5.8	0.5 ±0.0	18.6 ±2.6	51.2 ±13.6	8.4 ±2.6	113.8 ±8.9	9.8 ±0.2	7.6 ±0.5	0.3 ±0.5	258.0 ±74.2	9.6 ±5.0	145.4 ±1.0	4.5 ±0.3	106.4 ±1.2	678.9 ±155.3
**Fumed** **SAS**	5.8 ±0.3	3.8 ±0.2	1.9 ±0.1	0.0 ±0.0	380.0 ±81.9	97.0 ±19.0	27.8 ±3.4	0.5 ±0.1	15.9 ±1.7	55.2 ±8.4	8.0 ±1.7	112.4 ±11.2	9.8 ±0.1	7.6 ±0.6	0.1 ±0.3	378.0 ±140.2	8.8 ±4.0	144.2 ±0.7	4.5 ±0.2	105.8 ±1.2	762.9 ±178.1
**Precipitated SAS**	5.8 ±0.2	3.8 ±0.1	1.9 ±0.1	0.0 ±0.0	460.1 ±108.1	83.3 ±2.6	25.0 ±2.2	0.5 ±0.0	20.7 ±2.2	51.4 ±11.1	8.3 ±3.8	111.7 ±6.4	9.6 ±0.2	8.1 ±0.3	0.4 ±0.5	198.0 ±25.1	7.7 ±3.6	146.0 ±1.5	4.6 ±0.4	106.9 ±0.7	683.8 ±213.2

No significant differences among non-treated control, fumed SAS-treated, and precipitated SAS-treated group were found (*p* > 0.05). TP, total protein; ALB, albumin; A/G, albumin/globulin ratio; T-BIL, total bilirubin; ALP, alkaline phosphatase; AST, aspartate aminotransferase; ALT, alanine aminotransferase; CREA, creatine; BUN, blood urea nitrogen; T-CHO, total cholesterol; TG, triglycerides; GLU, glucose; CA, calcium; IP, inorganic phosphorus; GGT, Gamma glutamyl-transpeptidase; CK, creatine kinase; TBA, bile acid; Na^+^, sodium; K^+^, potassium; Cl^−^, chloride; CHE, cholinesterase.

**Table 6:** Histopathological findings of rats after 28-d repeated oral administration of fumed SAS and precipitated SAS in rats.

Organs	Number of Animals	Histopathological Findings	Group		
			Control	Fumed SAS	Precipitated SAS
Liver	5	No abnormality detected	4	4	5
		Inflammatory cell foci			
		-minimal, multifocal	0	1	0
		Vacuolation, periportal	1	0	0
Kidney	5	No abnormality detected	4	2	3
		Basophilia, tubular			
		-minimal	1	0	0
		-slight	0	0	1
		-slight, focal	0	1	0
		Cyst			
		-medullary, present	0	2	1
		Scar			
		-cortical, present	0	1	0
Heart	5	No abnormality detected	5	5	5
Lung	5	No abnormality detected	5	5	5
Brain	5	No abnormality detected	5	5	5
Spleen	5	No abnormality detected	5	5	5
Ovary	5	No abnormality detected	5	5	5
Stomach	5	No abnormality detected	5	5	4
		Cyst			
		-squamous, present	0	0	1
Small/large intestine	5	No abnormality detected	5	5	5
Pancreas	5	No abnormality detected	5	5	5

## Data Availability

The data presented in this study are available in the article and Supplementary Materials.

## Supplementary Materials

The following are available online at https://www.mdpi.com/article/10.3390/ijms23074023/s1.

## References

[1] Dera M.W.W., Teseme W.B. (2020). Review on the application of food nanotechnology in food processing. Am. J. Eng. Technol. Manag..

[2] Fruijtier-Polloth C. (2016). The safety of nanostructured synthetic amorphous silica (SAS) as a food additive (E 551). Arch. Toxicol..

[3] Younes M., Aggett P., Aguilar F., Crebelli R., Dusemund B., Filipič M., Frutos M.J., Galtier P., Gott D., Gundert-Remy U. (2018). Re-evaluation of silicon dioxide (E 551) as a food additive. EFSA J..

[4] Maharana S.M., Pandit M.K., Pradhan A.K. (2020). Effect of chemical treatment and fumed silica coating on tensile and thermogravimetric properties of jute yarn. Mater. Today Proc..

[5] Isfort C.S., Rochnia M. (2009). Production and physico-chemical characterisation of nanoparticles. Toxicol. Lett..

[6] Hewitt N., Ebnesajjad S. (2007). Silica as a reinforcing filler. Compounding Precipitated Silica in Elastomers.

[7] Musić S., Filipović-Vinceković N., Sekovanić L. (2011). Precipitation of amorphous SiO_2_ particles and their properties. Braz. J. Chem. Eng..

[8] Commision of the European Communities Food-Science and Techniques: Reports of the Scientific Committee for Food (Twenty-Fifth Series). http://aei.pitt.edu/40834/1/25th_food.pdf.

[9] U.S. Food and Drug Administration (FDA) CFR-Code of Federal Regulations Title 21.

[10] Bernardos Bau A., Kourlimska L. (2013). Applications of mesoporous silica materials in food. Czech J. Food Sci..

[11] Martin K.R. (2007). The chemistry of silica and its potential health benefits. J. Nutr. Health Aging.

[12] Dekkers S., Krystek P., Peters R.J., Lankveld D.P., Bokkers B.G., van Hoeven-Arentzen P.H., Bouwmeester H., Oomen A.G. (2011). Presence and risks of nanosilica in food products. Nanotoxicology.

[13] Van Kesteren P.C., Cubadda F., Bouwmeester H., van Eilkeren J.C., Dekkers S. (2014). Novel insights into the risk assessment of the nanomaterial synthetic amorphous silica, additive E551, in food. Nanotoxicology.

[14] Thakur R., Singh S., Kanchi S., Ahmed S. (2018). Nanosilica particles in food: A case of synthetic amorphous silica. Nanomaterials: Biomedical, Environmental, and Engineering Applications.

[15] Ogawa T., Okumura R., Nagano K., Minemura T., Izumi M., Motooka D., Nakamura S., Iida T., Maeda Y., Kumanogoh A. (2021). Oral intake of silica nanoparticles exacerbates intestinal inflammation. Biochem. Biophys. Res. Commun..

[16] Deng Y.D., Zhang X.D., Yang X.S., Huang Z.L., Wei X., Yang X.F., Liao W.Z. (2021). Subacute toxicity of mesoporous silica nanoparticles to the intestinal tract and the underlying mechanism. J. Hazard. Mater..

[17] Du Q., Ge D., Mirshafiee V., Chen C., Li M., Xue C., Ma X., Sun B. (2019). Assessment of neurotoxicity induced by differentsized Stöber silica nanoparticles: Induction of pyroptosis in microglia. Nanoscale.

[18] Wu J., Wang C., Sun J., Xue Y. (2011). Neurotoxicity of silica nanoparticles: Brain localization and dopaminergic neurons damage pathways. ACS Nano.

[19] Peters R.J., Oomen A.G., van Bemmel G., van Vliet L., Undas A.K., Munniks S., Bleys R.L.A.W., Tromp P.C., Brand W., van der Lee M. (2020). Silicon dioxide and titanium dioxide particles found in human tissues. Nanotoxicology.

[20] Gmoshinski I.V., Shipelin V.A., Shumakova A.A., Trushina E.N., Mustafina O.K., Safenkova I.V., Khotimchenko S.A., Nikityuk D.B., Tutelyan V.A. (2020). Toxicity evaluation of nanostructured silica orally administered to rats: Influence on immune system function. Nanomaterials.

[21] Brand W., van Kesteren P.C.E., Peters R.J.B., Oomen A.G. (2021). Issues currently complicating the risk assessment of synthetic amorphous silica (SAS) nanoparticles after oral exposure. Nanotoxicology.

[22] Yoo N.K., Jeon Y.R., Choi S.J. (2021). Determination of two differently manufactured silicon dioxide nanoparticles by cloud point extraction approach in intestinal cells, intestinal barriers and tissues. Int. J. Mol. Sci..

[23] Tran T.N., Anh Pham T.V., Phung Le M.L., Thoa Nguyen T.P., Tran V.M. (2013). Synthesis of amorphous silica and sulfonic acid functionalized silica used as reinforced phase for polymer electrolyte membrane. Adv. Nat. Sci. Nanosci. Nanotechnol..

[24] Paek H.J., Chung H.E., Lee J.A., Kim M.K., Lee Y.J., Kim M.S., Kim S.H., Maeng E.H., Lee J.K., Jeong J. (2014). Quantitative determination of silica nanoparticles in biological matrices and their pharmacokinetics and toxicokinetics in rats. Sci. Adv. Mater..

[25] Kim Y.R., Lee S.Y., Lee E.J., Park S.H., Seong N.W., Seo H.S., Shin S.S., Kim S.J., Meang E.H., Park M.K. (2014). Toxicity of colloidal silica nanoparticles administered orally for 90 days in rats. Int. J. Nanomed..

[26] Yu J., Choi S.J. (2021). Particle size and biological fate of ZnO do not cause acute toxicity, but affect toxicokinetics and gene expression profiles in the rat livers after oral administration. Int. J. Mol. Sci..

[27] Kim M.K., Lee J.A., Jo M.R., Choi S.J. (2016). Bioavailability of silica, titanium dioxide, and zinc oxide nanoparticles in rats. J. Nanosci. Nanotechnol..

[28] Lee J.A., Kim M.K., Song J.H., Jo M.R., Yu J., Kim K.M., Kim Y.R., Oh J.M., Choi S.J. (2017). Biokinetics of food additive silica nanoparticles and their interactions with food components. Colloids Surf. B Biointerfaces.

[29] Wu T., Tang M. (2018). Review of the effects of manufactured nanoparticles on mammalian target organs. J. Appl. Toxicol..

[30] Di Cristo L., Oomen A.G., Dekkers S., Moore C., Rocchia W., Murphy F., Johnston H.J., Janer G., Haase A., Stone V. (2021). Grouping hypotheses and an integrated approach to testing and assessment of nanomaterials following oral ingestion. Nanomaterials.

[31] Geraets L., Oomen A.G., Krystek P., Jacobsen N.R., Wallin H., Laurentie M., Verharen H.W., Brandon E.F., de Jong W.H. (2014). Tissue distribution and elimination after oral and intravenous administration of different titanium dioxide nanoparticles in rats. Part. Fibre Toxicol..

[32] Singh R., Lillard J.W. (2009). Nanoparticle-based targeted drug delivery. Exp. Mol. Pathol..

[33] Balaji E.V., Selvan A. (2019). Nanopharmacology: A novel approach in therapeutics. Asian J. Pharm. Sci..

[34] Lee J.A., Kim M.K., Paek H.J., Kim Y.R., Kim M.K., Lee J.K., Jeong J., Choi S.J. (2014). Tissue distribution and excretion kinetics of orally administered silica nanoparticles in rats. Int. J. Nanomed..

[35] Scientific Committee on Consumer Safety (SCCS) Opinion on Silica, Hydrated Silica, and Silica Surface Modified with Alkyl Silylates (Nano Form). https://ec.europa.eu/health/scientific_committees/consumer_safety/docs/sccs_o_175.pdf.

[36] Boorman G., Suttie A., Leininger J., Eustis S., Elwell M., Bradley A., Mackenzie W. (2017). Boorman’s Pathology of the Rat: Reference and Atlas.

[37] Whalan J.E. (2015). A Toxicologist’s Guide to Clinical Pathology in Animals: Hematology, Clinical Chemistry, Urinalysis.

[38] Yoo N.K. (2021). Comparative Study on Physicochmical Properties, Dissolution and Toxicokinetics of Differently Manufactured Food Additive Silicon Dioxide Nanoparticles. Master’s Thesis.

