# Supplementary material for: Oral Toxicokinetics, Tissue Distribution, and 28-Day Oral Toxicity of Two Differently Manufactured Food Additive Silicon Dioxides
Source: Int J Mol Sci. 2022 Apr 5;23(7):4023. doi: 10.3390/ijms23074023 (PMC8999665; doi:10.3390/ijms23074023)
Supplement: Supplementary file 1 [file ijms-23-04023-s001.zip › ijms-1630419-supplementary.pdf]

Oral toxicokinetics, tissue distribution, and 28-day oral toxicity of two differently manufactured food additive silicon dioxides

Na-Kyung Yoo, Su-Min Youn, and Soo-Jin Choi *

Division of Applied Food System, Major of Food Science & Technology, Seoul Women's University, Seoul 01797, Republic of Korea; iko0105@swu.ac.kr (N.-K.Y.); smyoun@swu.ac.kr (S.-M.Y.)

* Correspondence: sjchoi@swu.ac.kr; Tel.: +82-2-970-5634; Fax: +82-2-970-5977

Received: date; Accepted: date; Published: date

Supplementary Materials

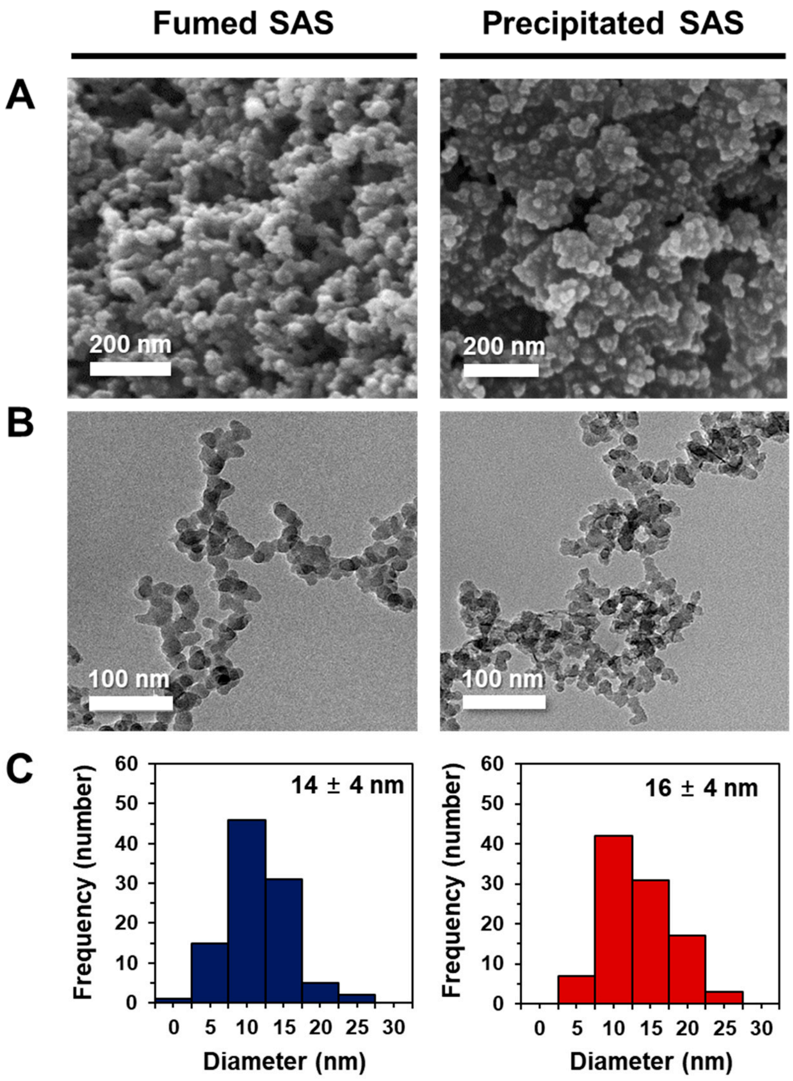

Figure S1. (A) Scanning electron microscopy (SEM), (B) transmission electron microscopy (TEM), and (C) size distributions of fumed SAS and precipitated SAS. Size distributions were measured by randomly selecting at least 100 particles from the TEM images.

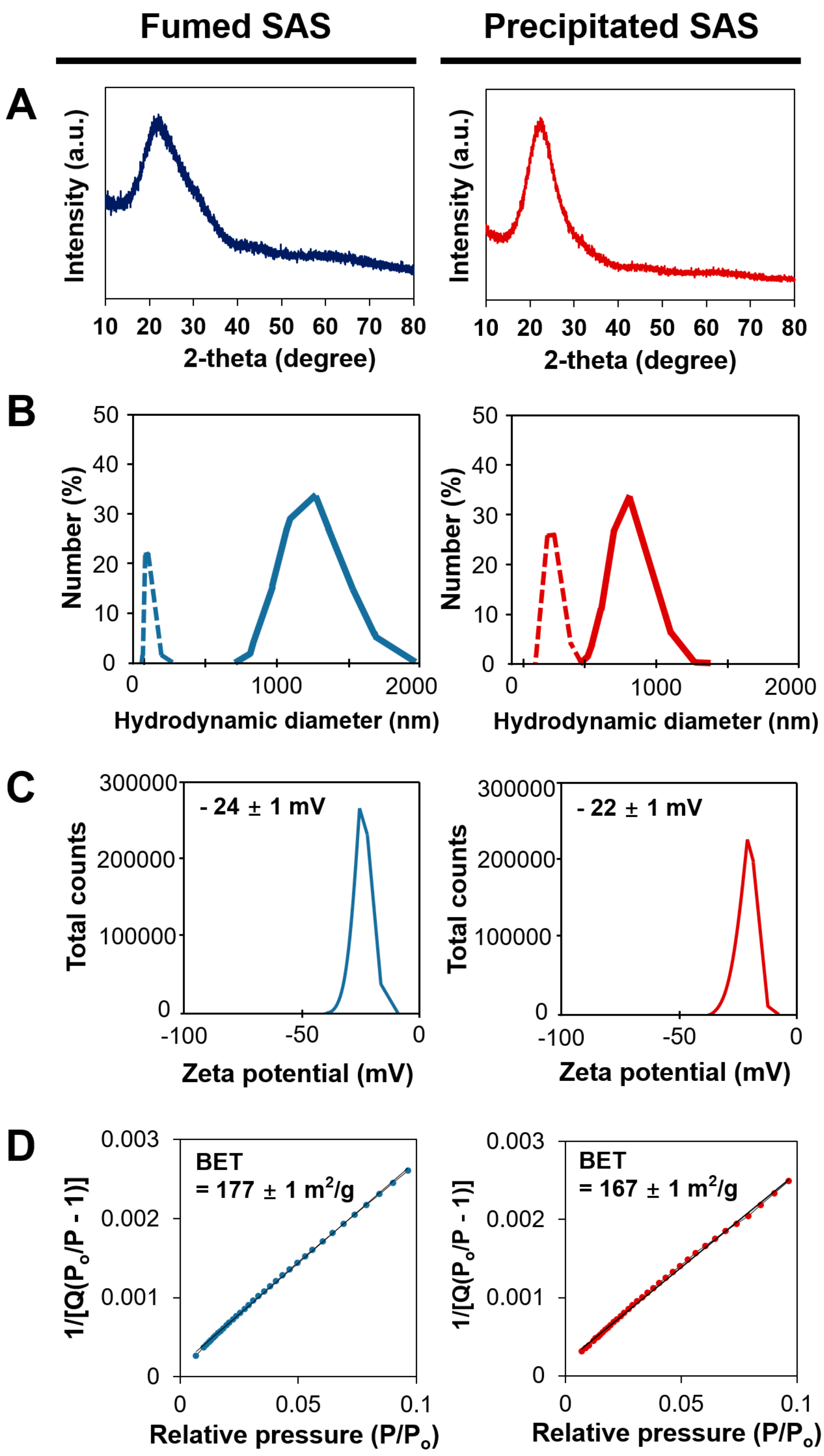

Figure S2. (A) X-ray diffraction (XRD) pattern, (B) hydrodynamic diameters in distilled water (DW, dotted line) and in vitro three consecutive steps of digestion fluids (thick solid line), (C) zeta potentials, and (D) Brunauer–Emmett–Teller (BET) specific surface areas of fumed SAS and precipitated SAS.

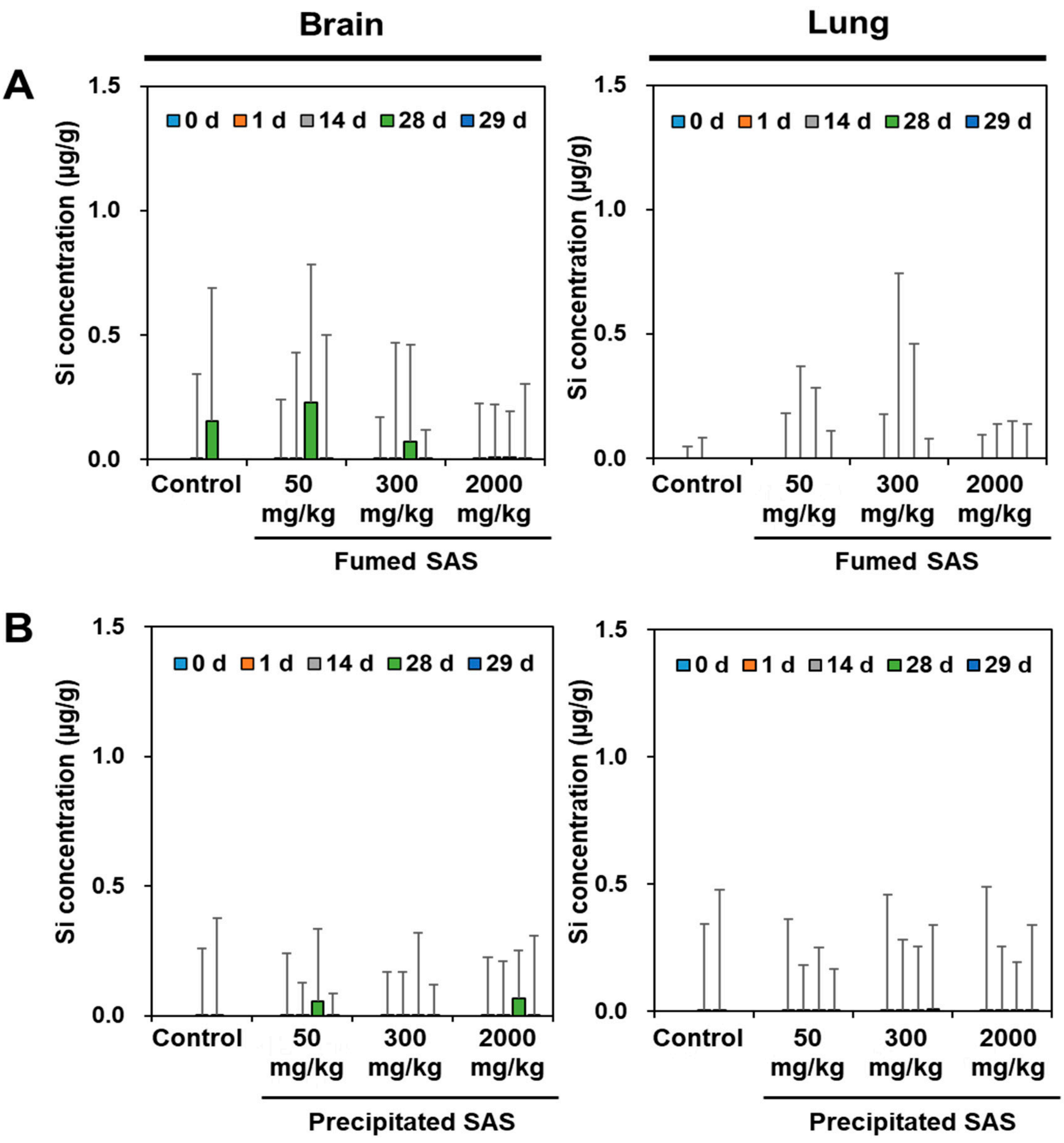

Figure S3. Tissue distributions of (A) fumed SAS and (B) precipitated SAS in the brain and lung after 28-d repeated oral administration in rats, followed by 90-d recovery period. No significant differences among non-treated control, fumed SAS-treated, and precipitated SAS-treated groups were found ($P > 0.05$).
